# The Co‐Occurrence of Autism and Avoidant/Restrictive Food Intake Disorder (ARFID): A Prevalence‐Based Meta‐Analysis

**DOI:** 10.1002/eat.24369

**Published:** 2025-01-06

**Authors:** Michelle Sader, Annabel Weston, Kyle Buchan, Jess Kerr‐Gaffney, Karri Gillespie‐Smith, Helen Sharpe, Fiona Duffy

**Affiliations:** ^1^ School of Medicine, Medical Sciences and Nutrition University of Aberdeen Aberdeen UK; ^2^ The Eating Disorders and Autism Collaborative (EDAC) University of Edinburgh Edinburgh UK; ^3^ School of Health in Social Science University of Edinburgh Edinburgh UK; ^4^ Institute of Psychiatry, Psychology and Neuroscience King's College London London UK; ^5^ NHS Lothian Child and Adolescent Mental Health Services Edinburgh UK

**Keywords:** ARFID, autism, avoidant/restrictive food intake disorder, co‐occurrence, meta‐analysis, prevalence

## Abstract

**Objective:**

Avoidant/restrictive food intake disorder (ARFID) is a feeding and eating disorder characterized by extensive avoidance and/or restriction of food. Existing research demonstrates that ARFID is over‐represented in Autistic populations and vice‐versa, with both groups exhibiting shared characteristics. This meta‐analysis investigated the co‐occurrence between ARFID and autism via determination of autism prevalence in ARFID populations, and ARFID prevalence in Autistic groups.

**Method:**

This review systematically identified literature evaluating those with ARFID and Autistic individuals. Literature was searched for using SCOPUS, MEDLINE, and Web of Science. Selected publications included Autistic individuals and those with ARFID who either received a formal diagnosis of autism and/or ARFID or met clinical threshold cut‐off scores on validated autism and/or ARFID questionnaires. Prevalence was reported in proportion‐based values alongside 95% confidence intervals (CIs).

**Results:**

This meta‐analysis identified 21 studies (kARFID = 18 papers; kAutism = 3 papers) comprising of *n* = 7442 participants (nARFID = 1708; nAutism = 5734). Prevalence of autism diagnoses was 16.27% in those with ARFID (95% CI = 8.64%–28.53%), and ARFID prevalence in Autistic groups was 11.41% (95% CI = 2.89%–35.76%). Gender and ethnicity served as significant sources of heterogeneity in ARFID papers. There was insufficient data to provide comparator values or prevalence across study population and distinct underpinning drivers of ARFID.

**Discussion:**

Meta‐analytic findings highlight significant rates of co‐occurrence between autism and ARFID, suggesting that in clinical settings, it may be beneficial to consider screening Autistic individuals for ARFID and vice‐versa. Future research should further investigate co‐occurrence across ARFID profiles, gender, and ethnicity.

**Summary**

- A proportion of research highlights a significant diagnostic overlap between avoidant/restrictive food intake disorder (ARFID) and autism.
- Meta‐analysis of co‐occurrence identified an autism diagnostic prevalence of 16.27% in 18 publications with ARFID groups, and an ARFID prevalence of 11.41% in 3 studies with Autistic groups.
- ARFID and autism co‐occurrence prevalence serves to inform clinicians on supporting Autistic individuals with disordered eating and tailoring existing eating disorder interventions.

## 1 Introduction

Avoidant/restrictive food intake disorder (ARFID) is a feeding and eating disorder (FED) introduced in the Diagnostic Statistical Manual of Mental Disorders, Fifth Edition (DSM‐5) in 2013 (American Psychiatric Association 2013). ARFID is primarily characterized by restriction or avoidance of food to the extent with which an individual's weight, nutritional intake, or psychosocial functioning are significantly impacted. Importantly, diagnostic criteria for ARFID state that the eating behaviors cannot be explained by a lack of food availability and cultural or religious practice, and is not primarily instantiated by body image disturbances or other health conditions (American Psychiatric Association 2013). ARFID is reported as clinically distinct from other feeding disorders or restrictive eating disorders (EDs) such as anorexia nervosa (AN; Fisher et al. 2014; Sharp and Stubbs 2019; Cañas et al. 2021). Diagnostic criteria identify different underpinning drivers for ARFID including 1. Lack of interest in food (LOF); 2. Sensory sensitivity associated with food (SS); 3. Fear of aversive consequences (FOC) associated with eating, such as choking or vomiting (Norris et al. 2018; American Psychiatric Association 2022). These are sometimes referred to as “subtypes” within the literature, but it is increasingly acknowledged that they are not distinct and can co‐occur (Sanchez‐Cerezo et al. 2024) and vary in severity, representing a more dimensional model (Thomas et al. 2017). ARFID is comorbid with a large range of psychiatric disorders including generalized anxiety (Eddy et al. 2015; Hay et al. 2017; Brigham et al. 2018; Zickgraf et al. 2019; Kambanis et al. 2020; Zanna et al. 2021), major depressive disorders/mood disorders (Zickgraf et al. 2019; Kambanis et al. 2020; Zanna et al. 2021), and OCD (Fisher et al. 2014; Nicely et al. 2014; Kambanis et al. 2020; Cañas et al. 2021), as well as physiological conditions including functional dyspepsia, irritable bowel syndrome and gastroparesis (Burton Murray et al. 2020).

ARFID also expresses significant rates of co‐occurrence with autism, a neurodevelopmental condition represented as a distinct neurotype characterized by differences in social communication, cognitive thinking styles, and information processing (American Psychiatric Association 2013). Due to extensive evidence that a significant proportion of Autistic individuals prefer identity‐first (e.g., Autistic person) over person‐first (e.g., person with autism) language when being referenced (Buijsman, Begeer, and Scheeren 2023; Bury et al. 2023; Taboas, Doepke, and Zimmerman 2023), this work will adhere to these language preferences when referring to the Autistic community, including capitalization of Autistic identity markers (Pineo 2022). Those with ARFID and Autistic individuals share multiple characteristics in relation to eating and feeding, such as sensory sensitivities, food selectivity, and lack of dietary diversity (Sharp et al. 2013; Zimmerman and Fisher 2017; Chistol et al. 2018; Sharp and Stubbs 2019; Islam et al. 2020; Li et al. 2022; Nimbley et al. 2022; Valenzuela‐Zamora, Ramírez‐Valenzuela, and Ramos‐Jiménez 2022; Watts et al. 2023). There is a notable proportion of autism diagnoses and/or demonstration of high levels of Autistic traits in those with ARFID (Nicely et al. 2014; Kambanis et al. 2020; Inoue et al. 2021; Sanchez‐Cerezo et al. 2023; Watts et al. 2023), as well as reports of elevated rates of ARFID in Autistic groups (Koomar et al. 2021; Nygren et al. 2021). Research also highlights increased familial susceptibility to ARFID, with 17% of parents of Autistic children at heightened risk for presenting with or developing ARFID (Koomar et al. 2021). The current evidence base suggests that research evaluating the co‐occurrence between autism and ARFID may shed light on the clinical manifestations and underlying mechanisms driving ARFID, and may inform on existing treatment options for the FED. However, despite burgeoning reports detailing an overrepresentation of ARFID diagnoses in Autistic individuals and vice‐versa, there has been no study systematically reviewing and quantifying the co‐occurrence of ARFID and autism.

This meta‐analysis aims to quantify the co‐occurring prevalence of autism diagnoses and/or elevated trait presence in ARFID, as well as ARFID diagnoses and/or elevated ARFID symptom presence in autism. This meta‐analysis additionally aims to determine the prevalence of ARFID and autism in control populations to report as comparator values. Where possible, this review also aims to quantify prevalence of co‐occurrence across study setting (e.g., clinical ED versus population‐based sample) and between the three primary underpinning drivers of ARFID (LOF, SS, FOC) in Autistic populations and vice‐versa.

## 2 Method

### 2.1 Eligibility Criteria

Studies investigating Autistic groups and studies investigating those with ARFID were included for this meta‐analysis. Autistic individuals and those with ARFID must have either received a formal diagnosis or met clinical threshold cut‐off scores for validated questionnaires evaluating autism (e.g., the autism quotient [AQ; Allison, Auyeung, and Baron‐Cohen 2012], broad autism phenotype questionnaire [BAPQ; Hurley et al. 2007], etc.) or ARFID (e.g., the Nine‐Item ARFID Screen [NIAS; Zickgraf and Ellis 2018]). This meta‐analysis also included studies with quantitative approaches utilizing primary (e.g., cross‐sectional designs) or secondary (e.g., secondary analysis of clinical data) analyses, providing the literature contained reports on ARFID/autism co‐occurrence. Exclusion criteria for publications during literature selection were as follows: 1. Papers were not in English; 2. Data were unavailable/inaccessible; 3. Papers consisted of reviews/meta‐analyses, qualitative studies, or case reports/pilot studies/opinion pieces; 4. ARFID and/or autism groups were not reported as distinct groups. Literature selection also included preprint studies and gray literature, providing papers fell under the aforementioned inclusion criteria.

### 2.2 Search Strategy and Selection of Literature

Literature was searched by two reviewers on March 9, 2024 using SCOPUS, Web of Science (WoS), and MEDLINE using identical search criteria. For all research databases, search terminology comprised of the following: (“ARFID” OR “avoidant restrictive food intake disorder*”) AND (“autis*” OR “asperger*” OR “pervasive development* disorder*”). As the formal inclusion of the ARFID diagnosis within the DSM‐5 occurred in 2013, searches were conducted from 2010 to the current date. Literature search was supplemented by searching gray literature using Google Scholar and EThOS, preprint servers such as PsyArXiv, SocArXiv, Medrxiv and Biorxiv, and the ProQuest Dissertations and Theses repository. Literature searches were re‐run on October 25, 2024 prior to the final analysis to include any recent relevant publications.

### 2.3 Study Selection and Data Extraction

Literature screening and data extraction were conducted using Covidence (https://www.covidence.org). For first‐ and second‐stage screening, two researchers independently reviewed the title and abstract, as well as the full text of identified publications, with a minimum of 30% of material independently reviewed. As the Covidence software does not blind users to information associated with publications, reviewers were aware of the authors and institutions associated with each study undergoing review. Any disagreements between reviewers were resolved via a third reviewer. Data were independently extracted and was exported as CSV sheets, including: 1. The number of individuals meeting criteria for ARFID; 2. The number of Autistic individuals across studies; 3. The number of individuals meeting criteria for ARFID profiles; 4. The number of individuals with no diagnosis of ARFID and/or autism. Extracted data also included information regarding the author, type of study, date of publication, methodological approach, participant sample size, participant characteristics, study environment, method of ARFID/autism classification/diagnosis, and rates of prevalence. This meta‐analysis was conducted in accordance with the 2020 Preferred Reporting Items for Systematic Reviews and Meta‐Analyses (PRISMA) guidelines. Prior to the initiation of this study, the protocol for this meta‐analysis was pre‐registered on PROSPERO and may be read here: crd.york.ac.uk/prospero/display_record.php?ID=CRD42024517036.

### 2.4 Risk of Bias Assessment

Post‐extraction and screening of literature, included publications were assessed for quality and risk of bias using the Joanna Briggs Institute (JBI) checklist (Munn et al. 2014) for prevalence studies to examine the suitability and validity of included publications. Two researchers assessed included publications for quality and risk of bias. From all included publications, 30% were independently appraised for risk of bias, with 70% of studies collaboratively appraised. Any disagreements that could not be resolved between reviewers were resolved using a third reviewer.

### 2.5 Data Analysis and Synthesis

All statistical analysis was done using the R Software (Version 4.4.1; R Core Team 2024). Random‐effects model meta‐analyses were performed using the “meta” package on the R software (https://cran.r‐project.org/web/packages/meta/meta.pdf). To assess group‐specific proportions and odds ratios, two meta‐analyses were performed in parallel across publications reporting on autism and ARFID. A generalized linear mixed model (GLMM) approach was utilized to stabilize the variance of proportions across individual studies. The GLMM approach was utilized as this method less susceptible to issues with back transformation of data than alternative approaches such as the Freeman–Tukey double arcsine transformation (Freeman and Tukey 1950) and is more appropriate for use in meta‐analyses incorporating random‐effects methods (Schwarzer et al. 2019; Lin and Xu 2020). Clopper–Pearson intervals (Clopper and Pearson 1934) were used to calculate confidence intervals for individual studies. For each outcome, meta‐analyses generated respective findings on proportions, presented as a proportion of (nAutism/nARFID) in ARFID studies, (nARFID/nAutism) for studies with Autistic groups and (nAutism OR nARFID/Total Study Population) alongside 95% confidence intervals. Heterogeneity of included publications was reported via Tau^2^, *I* ^2^, and *H* ^2^ values (Higgins and Thompson 2002). Tau^2^ is a point‐based estimate of true‐effect study variance, with values closer to zero indicating lower variability between studies (West et al. 2011). *I* ^2^ is a measurement of the proportion derived from Tau^2^ point estimates, with 0%–25% indicating low heterogeneity, and 75%–100% indicating high heterogeneity (Higgins and Thompson 2002; West et al. 2011). *H* ^2^ is a measure comparing observed variation in estimates relative to what would be expected if all studies were estimating identical effects, with values higher than 1 indicate increasing heterogeneity that is larger than what would be expected by chance (Higgins and Thompson 2002; West et al. 2011). It is recommended to provide both *H* ^2^ and *I* ^2^ statistics to provide a diverse assessment of heterogeneity (Higgins and Thompson 2002), but the Tau^2^ statistic was included when reporting findings as this measure is preferable relative to the *I* ^2^ statistic when meta‐analyses include a smaller number of studies (von Hippel 2015). The likelihood ratio test (LRT; Wilks 1938) was also used to assess heterogeneity across publications, with a significant *p* value for this statistic suggesting high heterogeneity across papers (Schwarzer, Carpenter, and Rücker 2015). Publication bias was assessed using Egger's tests (Egger et al. 1997) alongside funnel plots.

The majority of literature focusing on ARFID has been conducted in clinical ED settings (Duffy et al. 2024), which may serve to impact the generalizability of recent ARFID‐related findings to the broad, population‐based proportion of people experiencing the FED. Providing availability of data relating to study environment within included literature, this meta‐analysis seeks to conduct subgroup‐based analyses evaluating rates of co‐occurrence according to study setting. Providing availability of comparator data, an identical approach would be taken to determine prevalence of ARFID/autism respectively in control populations to report as comparator values. Comparators were presented as respective odds ratios calculated by ([nARFID/nAutism]/[nARFID/nNeurotypical]) for studies with Autistic samples, and ([nAutism/nARFID]/[nAutism/nHealthy Control]) for ARFID studies. Providing sufficient data, this meta‐analysis aims to conduct subgroup analyses using an identical methodological approach based on the presentation of ARFID profiles (LOF, SS, FOC). For all analyses, between‐study heterogeneity was assessed using a jackknife analysis, a methodological approach that involves repeated assessment of data under identical parameters upon one‐by‐one removal of studies (Miller 1974; Efron 1982). Mixed‐effects model meta‐regression analyses were also conducted as an exploratory analysis to identify potential sources of heterogeneity across publications including factors such as date of publication, study environment, method of ARFID and autism diagnosis, sample size, gender, age, and ethnicity.

## 3 Results

### 3.1 Sociodemographic Characteristics

**FIGURE 1:**
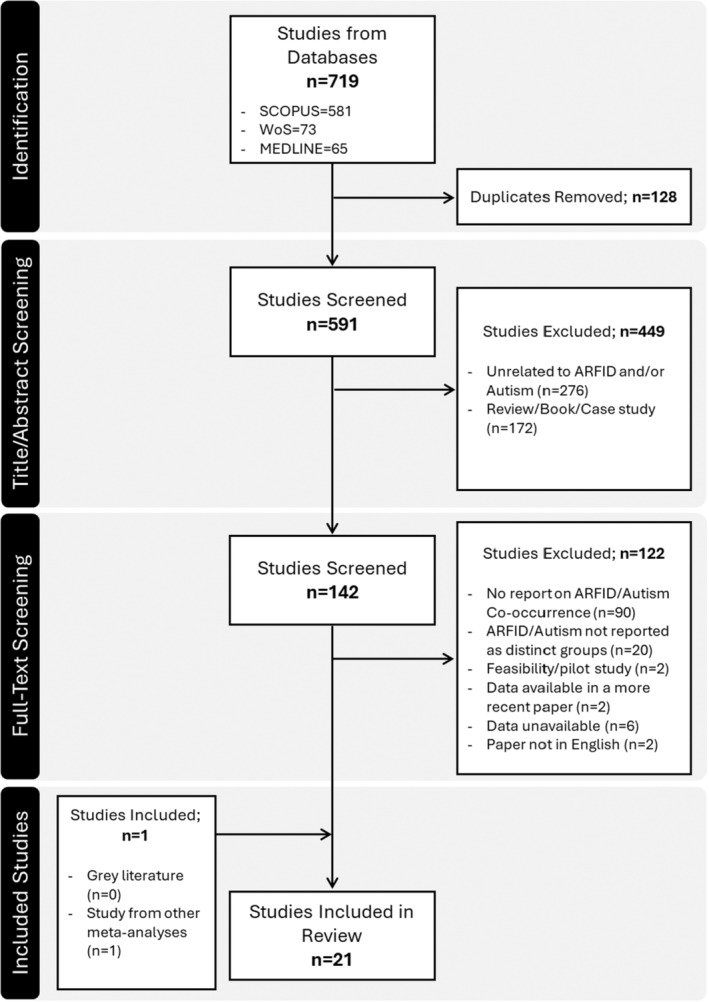
PRISMA flowchart depicting the study search and selection process. ARFID—avoidant/restrictive food intake disorder.

An initial search contained 719 papers from all databases, published between 2010 and 2024. A total of 128 duplicate publications were omitted from screening, leaving 591 publications available for a title and abstract screening. Title/abstract screening led to the removal of 449 publications, with 142 studies identified for a full‐text review. No additional papers in the form of gray literature were identified via Google Scholar, EThOS, preprint servers (PsyArXiv, SocArXiv, Medrxiv, and Biorxiv) or the ProQuest Dissertations and Theses repository as meeting eligibility criteria for this meta‐analysis, but one publication was identified from other identified meta‐analyses (Wong, Goh, and Ramachandran 2022). In total, 21 publications were selected for this meta‐analysis, with 18 publications (Nicely et al. 2014; Gray et al. 2018; Lange et al. 2019; Duncombe Lowe et al. 2019; Kambanis et al. 2020; Cañas et al. 2021; Inoue et al. 2021; Volkert et al. 2021; Dinkler et al. 2022; Farag et al. 2022; Katzman et al. 2022; Taylor and Taylor 2022; Wong, Goh, and Ramachandran 2022; Brosig et al. 2023; Chatoor et al. 2023; Watts et al. 2023; Bertrand et al. 2024; Sanchez‐Cerezo et al. 2024) focusing on studies with ARFID samples, and three publications (Sedgewick, Leppanen, and Tchanturia 2020; Koomar et al. 2021; Nygren et al. 2021) containing Autistic groups (Figure 1).

In total, the 21 studies assessed in this review included 1708 individuals with ARFID and 5734 Autistic individuals. The average age of ARFID participants was 11.44 ± 5.23 years, with one out of 18 papers containing an adult sample (Lange et al. 2019). The average age of Autistic participants was 16.11 ± 5.83 years, with one out of three papers investigating adults (Sedgewick, Leppanen, and Tchanturia 2020). The mean BMI for studies containing individuals with ARFID was 17.69 ± 2.83 in studies reporting via BMI values, and −1.14 ± 0.43 in studies reporting BMI‐for‐age *z* scores. Studies with Autistic groups did not report on BMI. The mean prevalence of gender presentation for ARFID and Autistic groups was 50.10% and 58.12% males, respectively (Table 1).

**TABLE 1:** Sociodemographic characteristics of all included studies (*k* = 21).

[ARFID papers] Author	Study type	Method of diagnosis	nTotal	nARFID	nOther	nARFID gender (% male)	ARFID age (*M* + SD); range	ARFID BMI (*M* + SD)	Mean ARFID DOI (Y)	Autism/ARFID Co‐Oc. (*n*; %)
Bertrand et al. 2024	Retrospective cohort study	ARFID: gastroent. + psychologist + ARFID module (Ch)EDE; autism: gastroent. + psychologist	190	100	57 UFED; 33 AN	60.0	5.7 (3.3); NA	NA (NA)	3.4 (NA)	7/100; 7.0
Sanchez‐Cerezo et al. 2024	Observational surveillance study	ARFID: DSM‐5 + researchers; autism: previous diagnosis	319	319	—	73.8	9.9 (3.7); NA	16.0 (2.4)	4.8 (3.6)	137/319; 42.95
Brosig et al. 2023	Observational cohort study	ARFID: clinician + ARFID module (Ch)EDE; autism: clinician	51	51	—	54.9	7.5 (5.4); NA	−1.5 (1.0)^a^	—	1/51; 2.0
Chatoor et al. 2023	Retrospective chart review	ARFID: clinician + DSM‐5; autism: clinician	62	32	30 HC	NA	NA (NA); NA	NA (NA)	—	2/32; 6.3
Watts et al. 2023	Clinical audit	ARFID: PARDI; autism: clinician	261	261	—	49.0	12.2 (4.1); 2.9–17.8	−1.1 (1.6)^a^	NA (NA)	74/261; 28.0
Taylor and Taylor 2022	Retrospective chart review	ARFID: physician; autism: previous diagnosis	31	32	—	75.0	6 (NA); 2.0–13.0	NA (NA)	—	28/32; 88.0
Dinkler et al. 2022	Prospective population study	ARFID: ARFID‐BS; autism: previous diagnosis	3728	49	3679 HC	44.9	5.6 (1.0); 4.2–7.3	NA (NA)	NA (NA)	4/49; 8.2
Farag et al. 2022	Prospective case–control study	ARFID: previous diagnosis; autism: previous diagnosis	536	263	273 Non‐ARFID	81.75	NA (NA); 0.0–10.0+	NA (NA)	—	144/26; 54.8
Katzman et al. 2022	Retrospective chart review	ARFID: pediatrician + DSM‐5; autism: pediatrician/psychiatrist + psychologist	207	207	—	38.6	13.1 (3.2); 9.0–18.0	−1.4 (1.2)^a^	—	17/207; 8.2
Wong, Goh, and Ramachandran 2022	Retrospective cohort study	ARFID: multidisciplinary ED team; autism: previous diagnosis	177	12	151 AN; 2 BN; 5 EDNOS; 7 ON	50.0	14.7 (2.1); NA	NA	NA (NA)	5/12; 41.7
Cañas et al. 2021	Observational comparative study	ARFID: previous diagnosis + DSM‐5; autism: previous diagnosis	108	42	33 AN; 33 HC	NA	NA (NA); NA	NA (NA)	NA (NA)	6/42; 9.1
Volkert et al. 2021	Retrospective cohort study	ARFID: dietitian + psychologist + DSM‐5; autism: clinician	60	60	—	83.0	6.0 (3.3); 1.9–15.1	−0.6 (1.5)^a^	5 (NA)	38/60; 63.0
Inoue et al. 2021	Prospective multicenter cohort study	ARFID: pediatrician + DSM‐5; autism: AQC + DSM‐5	124	32	92 AN	22.0	11.8 (2.4); NA	−3.2 (1.8)^b^	NA (NA)	4/32; 12.5
Kambanis et al. 2020	Observational cohort study	ARFID: PARDI; autism: KSADS‐PL	74	74	—	51.0	15.0 (3.5); 9.0–22.0	NA (NA)	—	2/74; 3.0
Duncombe Lowe et al. 2019	Cohort‐based study	ARFID: clinician + DSM‐5 + EDE‐Q^c^; autism: previous diagnosis	102	102	—	40.2	12.3 (2.7); NA	16.1 (2.7)	NA (NA)	6/102; 5.88
Lange et al. 2019	Retrospective chart review	ARFID: psychiatrist + physiotherapist + EDE‐Q^c^ + SWEAA^c^; autism: psychiatrist	56	19	37 AN	0.0	25.5 (5.4); 19.4–40.7	21.9 (3.3)	—	1/19; 5.0
Gray et al. 2018	Retrospective chart review	ARFID: clinician + DSM‐5; autism: previous diagnosis	14	14	—	57.0	15.2 (5.5); 7.0–23.0	16.8 (1.6)	NA (NA)	2/14; 14.3
Nicely et al. 2014	Retrospective chart review	ARFID: psychiatrist + DSM‐4‐TR + DSM‐5; autism: psychiatrist	173	39	93 AN; 20 BN; 21 UFED	20.5	11.1 (1.7); NA	NA (NA)	0.82 (1.1)	5/39; 13.0

[Autism papers] Author	Study type	Method of diagnosis	nTotal	nAutism	nOther	nAutism gender (% male)	Autism age (*M* + SD); range	Autism BMI (*M* + SD)	ARFID/autism Co‐Oc. (*n*; %)
Koomar et al. 2021	Cohort‐based study	ARFID: NIAS; autism: previous diagnosis + parent/caregiver or online survey	10,142	5157	4985 Parents	81.0	11.1 (5.9); NA	NA (NA)	1083/5157; 21.0
Nygren et al. 2021	Prospective longitudinal study	ARFID: pediatrician/psychiatrist + psychologist + DSM‐5; autism: pediatrician/psychiatrist + psychologist	46	46	—	80.0	3.2 (0.8); 1.8–4.9	NA (NA)	13/46; 28.0
Sedgewick, Leppanen, and Tchanturia 2020	Large‐scale online study	ARFID: previous diagnosis + EDE‐Q^c^; autism: previous diagnosis + AQ	930	531	399 HC	13.4	34.1 (10.9); 18.1–71.5	NA (NA)	11/531; 2.1

Abbreviations: AN—anorexia nervosa; AQ—autism quotient; AQC—autism quotient children's version; ARFID—avoidant/restrictive food intake disorder; ARFID‐BS—ARFID brief screener; BN—bulimia nervosa; Ch(EDE)—child eating disorder examination; Co‐Oc.—co‐occurrence; DOI—duration of illness; DSM‐4‐TR—diagnostic and statistical manual of mental disorders, 4th edition text revision; DSM‐5—diagnostic and statistical manual of mental disorders, 5th edition; EDE‐Q—eating disorder examination questionnaire; Gastroent.—gastroenterologist; HC—healthy control; K‐SADS‐PL—schedule for affective disorders and schizophrenia for school‐age children‐present and lifetime; M—mean; NA—not available; NIAS—nine‐item ARFID screen; ON—orthorexia nervosa; PARDI—pica, ARFID, and rumination disorder interview; SD—standard deviation; SWEAA—Swedish eating assessment of autism spectrum disorders; UFED—unspecified feeding and eating disorder; Y—years.

^a^ BMI measured via BMI‐for‐age *z* scores.

^b^ BMI measured via SD scores.

^c^ Questionnaire was used to investigate eating disorder characteristics, but was not used for a diagnosis of ARFID.

Within ARFID samples, 16 detected ARFID via clinical diagnosis or clinical interview (Nicely et al. 2014; Gray et al. 2018; Lange et al. 2019; Duncombe Lowe et al. 2019; Kambanis et al. 2020; Cañas et al. 2021; Inoue et al. 2021; Volkert et al. 2021; Farag et al. 2022; Katzman et al. 2022; Taylor and Taylor 2022; Wong, Goh, and Ramachandran 2022; Chatoor et al. 2023; Watts et al. 2023; Bertrand et al. 2024; Sanchez‐Cerezo et al. 2024), and 2 utilized previously published and validated questionnaires to identify likely ARFID (Dinkler et al. 2022; Brosig et al. 2023). From the three available publications evaluating the presence of ARFID in Autistic groups, all studies assessed for autism via clinical diagnosis or via previous medical history (Sedgewick, Leppanen, and Tchanturia 2020; Koomar et al. 2021; Nygren et al. 2021). Across all included studies, 16 recruited samples from clinical ED services (Nicely et al. 2014; Gray et al. 2018; Lange et al. 2019; Duncombe Lowe et al. 2019; Cañas et al. 2021; Inoue et al. 2021; Volkert et al. 2021; Farag et al. 2022; Katzman et al. 2022; Taylor and Taylor 2022; Wong, Goh, and Ramachandran 2022; Brosig et al. 2023; Chatoor et al. 2023; Watts et al. 2023; Bertrand et al. 2024; Sanchez‐Cerezo et al. 2024), and 5 recruited samples from the general population (Sedgewick, Leppanen, and Tchanturia 2020; Kambanis et al. 2020; Nygren et al. 2021; Koomar et al. 2021; Dinkler et al. 2022). Across all 21 included publications, 7 originated from the United States (US; Nicely et al. 2014; Gray et al. 2018; Duncombe Lowe et al. 2019; Kambanis et al. 2020; Koomar et al. 2021; Volkert et al. 2021; Chatoor et al. 2023), 4 originated from the United Kingdom (UK; Sedgewick, Leppanen, and Tchanturia 2020; Farag et al. 2022; Watts et al. 2023; Sanchez‐Cerezo et al. 2024), 2 originated from Sweden (Lange et al. 2019; Nygren et al. 2021), Japan (Inoue et al. 2021; Dinkler et al. 2022), and Germany/France (Brosig et al. 2023; Bertrand et al. 2024) respectively, and 1 publication from Australia (Taylor and Taylor 2022), Canada (Katzman et al. 2022), Singapore (Wong, Goh, and Ramachandran 2022), and Spain (Cañas et al. 2021), respectively.

### 3.2 Quality Assessment and Critical Appraisal

All included studies scored 5–9 out of 9 based on the JBI critical appraisal checklist (Munn et al. 2014) for prevalence‐based meta‐analyses (Table S1). All papers met criteria for items 6 (appropriateness of condition identification; 100%) and 8 (appropriateness of statistical analysis; 100%). Most of the included studies met items 2 (appropriateness of group sampling; 95.2%), 5 (sufficient coverage of sample during data analysis; 85.7%), and 7 (appropriateness of condition measurement; 95.2%). Only 61.9% of publications met criteria for item 3 (adequacy of sample size) as calculated via the following equation: *n* = *Z*2*p*(1 − *p*)/*d*2 (*n*—sample size; *Z*—standard normal distribution at a desired confidence interval; *p*—expected prevalence across individual studies; *d*—precision, corresponding to effect size; Munn et al. 2014). For item 4 (sufficient description of study setting and subjects; 66.6%), included publications did not meet criteria if no reports on participant ethnicity were available. The lowest criteria met was item 1 (appropriateness of sample frame; 23.8%), as studies including samples recruited from an ED clinic were not considered to be derived from an appropriate sample frame. For item 9, the response rate was not applicable for any publications conducting retrospective chart reviews.

### 3.3 Rates of Co‐Occurrence Between Autism and ARFID

**FIGURE 2:**
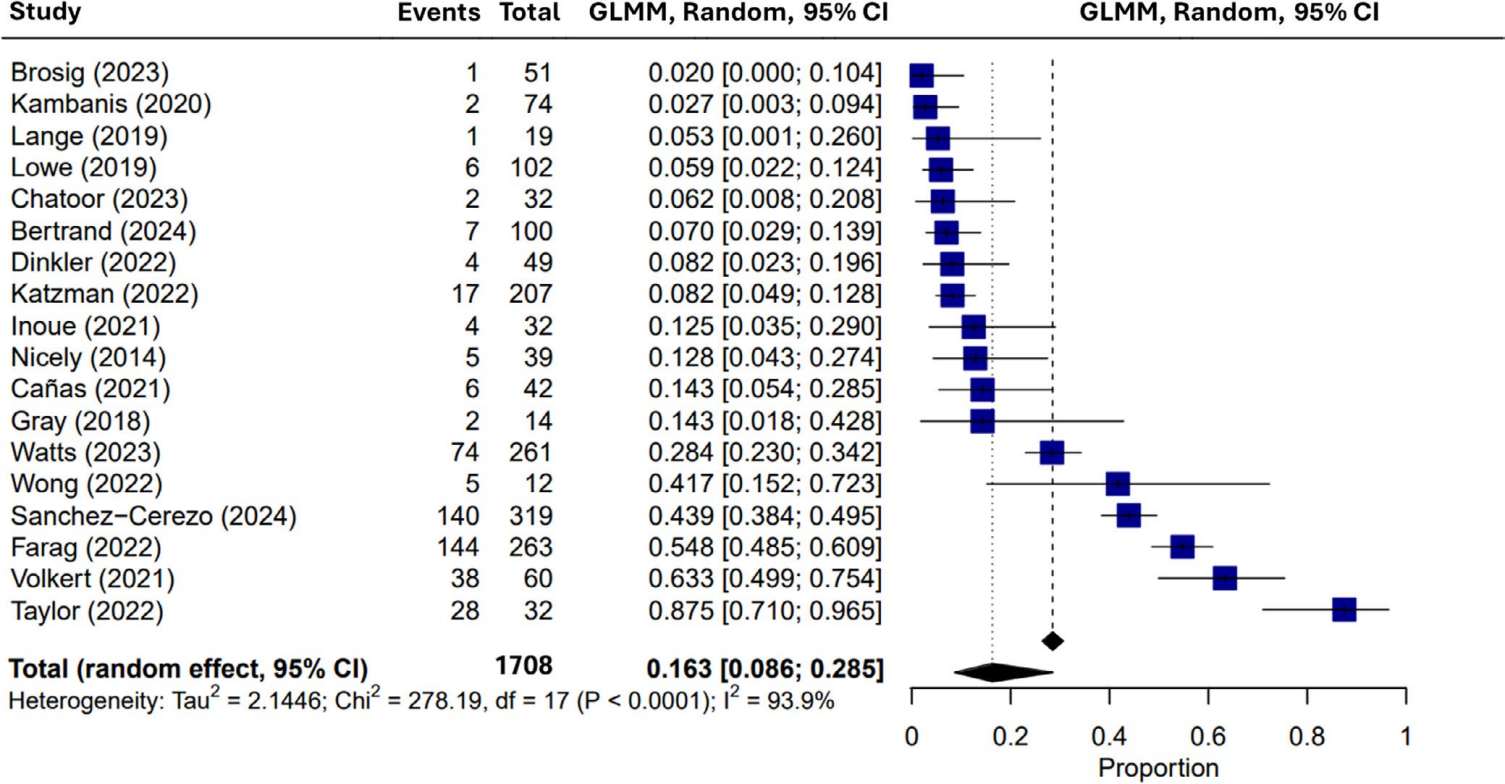
Meta‐analysis of proportions concerning autism prevalence across all ARFID studies (*k* = 18). CI—confidence interval; GLMM—generalized linear mixed model.

**FIGURE 3:**
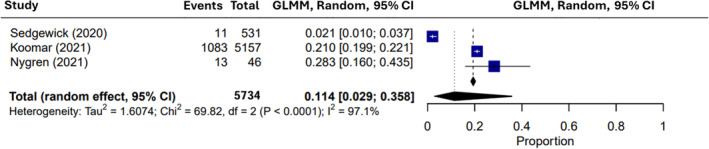
Meta‐analysis of proportions concerning ARFID prevalence across all autism studies (*k* = 3). CI—confidence interval; GLMM—generalized linear mixed model.

Within the 18 papers evaluating the prevalence of autism diagnoses in those with ARFID, the random‐effects meta‐analysis identified an average rate of 16.27% (95% confidence interval of 8.64% to 28.53%) co‐occurrence, ranging from 1.96% to 88.00% (Figure 2; Table 2). High heterogeneity was observed across publications, as seen by Tau^2^ (2.145), *H* ^2^ (4.05^2^ [3.47^2^, 4.72^2^]) and *I* ^2^ (93.9% [91.7%, 97.2%]) values, in addition to the presence of publication bias as seen by the Egger's test significance value (*p* = 0.0133; Figure S1). While only three publications were available to investigate the prevalence of ARFID in Autistic groups, meta‐analysis identified an average rate of 11.41% (95% confidence interval of 2.89%–35.76%) (Figure 3; Table 3). Higher heterogeneity was observed across these three publications relative to the included ARFID publications (Tau^2^ = 1.268; *H* ^2^ = 5.91^2^ [4.18^2^, 8.34^2^]; *I* ^2^ = 97.1% [94.3%, 98.6%]). There was insufficient literature to evaluate the prevalence of autism and/or ARFID in neurotypical or control groups as comparator values. Due to the limited availability of publications investigating rates of ARFID in studies with Autistic cohorts (*k* = 3), it is important to highlight factors across these publications that may contribute to the significant reported levels of heterogeneity. Out of all three studies, the publication by Sedgewick, Leppanen, and Tchanturia (2020) was conducted in an online‐only format, contained a significantly lower percentage of male participants and reported a significantly higher mean age than the other two publications (Table 1). While these differences may provide a potential explanation for the inconsistencies in ARFID prevalence across Autistic groups, there are too few studies to statistically assess these disparities. As a minimum of 10 studies are recommended to assess publication bias (Cumpston et al. 2019), there were also too few studies to reliably conduct Egger's tests and funnel plots in publications with Autistic groups. There was also insufficient literature to evaluate the prevalence of autism and/or ARFID in neurotypical or control groups as comparator values.

**TABLE 2:** Meta‐analysis of ARFID‐autism co‐occurrence (autism/ARFID; *k* = 18).

Author	Proportion	95% CI
Bertrand et al. 2024	0.0700	[0.0286, 0.1389]
Sanchez‐Cerezo et al. 2024	0.4389	[0.3836, 0.4952]
Brosig et al. 2023	0.0196	[0.0005, 0.1045]
Chatoor et al. 2023	0.0625	[0.0077, 0.2081]
Watts et al. 2023	0.2835	[0.2297, 0.3424]
Taylor and Taylor 2022	0.8750	[0.7101, 0.9649]
Dinkler et al. 2022	0.0816	[0.0227, 0.1960]
Farag et al. 2022	0.5475	[0.4852, 0.6087]
Katzman et al. 2022	0.0821	[0.0486, 0.1282]
Wong, Goh, and Ramachandran 2022	0.4167	[0.1517, 0.7233]
Cañas et al. 2021	0.1429	[0.0543, 0.2854]
Volkert et al. 2021	0.6333	[0.4990, 0.7541]
Inoue et al. 2021	0.1250	[0.0351, 0.2899]
Kambanis et al. 2020	0.0270	[0.0033, 0.0942]
Duncombe Lowe et al. 2019	0.0588	[0.0219, 0.1236]
Lange et al. 2019	0.0526	[0.0013, 0.2603]
Gray et al. 2018	0.1429	[0.0178, 0.4281]
Nicely et al. 2014	0.1282	[0.0430, 0.2743]
Average (fixed‐effect)	0.2845	[0.2636, 0.3064]
**Average (random‐effects)**	**0.1627**	**[0.0864, 0.2853]**
*I* ^2^ [CI]	93.90%	[91.70%, 97.20%]
Tau^2^ [CI]	2.145	
*H*	4.05^2^	[3.47^2^, 4.72^2^]
LRT *Q* (DF); *p*	417.26 (17); < 0.0001	
Egger's test; *p*	−3.62; *p* = 0.0133	

*Note*: Meta‐analyses were conducted using both fixed‐effect and random‐effects approaches. Values in bold text demonstrate that the random‐effects model was used to report proportions in this meta‐analysis.

Abbreviations: ARFID—avoidant/restrictive food intake disorder; CI—confidence interval; DF—degrees of freedom; LRT—likelihood ratio test; *Q*—Cochran's Q test.

**TABLE 3:** Meta‐analysis of ARFID‐autism co‐occurrence (ARFID/autism; *k* = 3).

Author	Proportion	95% CI
Koomar et al. 2021	0.2100	[0.1990, 0.2214]
Nygren et al. 2021	0.2826	[0.1599, 0.4346]
Sedgewick, Leppanen, and Tchanturia 2020	0.0207	[0.0104, 0.0368]
Average (fixed‐effect)	0.1931	[0.1738, 0.1942]
**Average (random‐effects)**	**0.1141**	**[0.0143, 0.3639]**
*I* ^2^ [CI]	97.1%	[94.3%, 98.6%]
Tau^2^ [CI]	1.268	
*H*	5.91^2^	[4.18^2^; 8.34^2^]
LRT *Q* (DF); *p*	163.66 (2); < 0.0001	
Egger's test; *p*	N/A	

*Note*: Meta‐analyses were conducted using both fixed‐effect and random‐effects approaches. Values in bold text demonstrate that the random‐effects model was used to report proportions in this meta‐analysis.

Abbreviations: ARFID—avoidant/restrictive food intake disorder; CI—confidence interval; DF—degrees of freedom; *Q*—Cochran's *Q* test.

Across all included studies, there was insufficient available data to conduct subgroup analyses based on study environment, as only 1 publication within ARFID groups was conducted in a population‐based sample (Dinkler et al. 2022), and all publications with Autistic groups were conducted in population‐based samples. There was also insufficient data to conduct subgroup analyses based on the presentation of LOF, SS, and FOC ARFID profiles. Two publications reported on the presence of autism diagnoses or elevated Autistic traits in those with an SS profile, at 48.94% (Sanchez‐Cerezo et al. 2024) and 21.42% (Katzman et al. 2022), respectively. Three publications reported on the presence of autism diagnoses or elevated Autistic traits in those with a LOF profile, at 26.25% (Sanchez‐Cerezo et al. 2024), 6.25% (Chatoor et al. 2023), and 12.85% (Katzman et al. 2022), respectively. Only one publication reported on the presence of autism diagnoses or elevated presence of Autistic traits in those with a FOC profile, at 13.04% (Sanchez‐Cerezo et al. 2024). No information on ARFID profile presentation was available for included publications with Autistic groups.

### 3.4 Sensitivity Analysis

Jackknife sensitivity analyses identified mean prevalence of autism diagnoses or presence of elevated Autistic traits in ARFID to range between 13.91% and 18.06%, depending one‐by‐one omission of publications. The mean prevalence was > 16.00% in 12/17 analyses. Prevalence was lowest at < 15.00% upon removal of Farag et al. (2022), Taylor and Taylor (2022), and Volkert et al. (2021) (Table 4). Contrasting the sensitivity analysis provided by the ARFID papers, publications with Autistic groups were highly heterogeneous and prevalence of ARFID in Autistic groups ranged widely from 8.07% to 21.06% depending on omission of publications (Table 5). The heterogeneity within Autistic studies relative to papers evaluating ARFID are likely distinct due to differences in sample size.

**TABLE 4:** Jackknife sensitivity analysis for ARFID papers (*k* = 18).

Author excluded	Proportion	Proportion [95% CI]	*I* ^2^	*I* ^2^ [95% CI]	*H* ^2^	*H* ^2^ [95% CI]	LRT (*Q*)	LRT *Q*; *p*
Bertrand et al. 2024	0.1710	[0.0886, 0.3046]	93.70%	[91.3%, 95.4%]	3.97^2^	[3.39^2^, 4.66^2^]	386.72	< 0.0001
Sanchez‐Cerezo et al. 2024	0.1504	[0.0774, 0.2721]	93.90%	[91.6%, 95.6%]	4.05^2^	[3.46^2^, 4.74^2^]	374.11	< 0.0001
Brosig et al. 2023	0.1806	[0.0977, 0.3096]	94.00%	[91.8%, 95.6%]	4.09^2^	[3.49^2^, 4.79^2^]	390.57	< 0.0001
Chatoor et al. 2023	0.1717	[0.0896, 0.3038]	94.10%	[91.9%, 95.7%]	4.11^2^	[3.51^2^, 4.80^2^]	406.96	< 0.0001
Watts et al. 2023	0.1558	[0.0792, 0.2838]	94.10%	[92.0%, 95.7%]	4.12^2^	[3.53^2^, 4.82^2^]	417.26	< 0.0001
Taylor and Taylor 2022	0.1391	[0.0791, 0.2333]	93.70%	[91.4%, 95.4%]	3.99^2^	[3.40^2^, 4.68^2^]	367.26	< 0.0001
Dinkler et al. 2022	0.1694	[0.0876, 0.3025]	94.00%	[91.8%, 95.6%]	4.08^2^	[3.48^2^, 4.77^2^]	404.49	< 0.0001
Farag et al. 2022	0.1472	[0.0768, 0.2638]	92.80%	[89.9%, 94.8%]	3.72^2^	[3.15^2^, 4.39^2^]	320.93	< 0.0001
Katzman et al. 2022	0.1693	[0.0872, 0.3033]	92.90%	[90.1%, 94.9%]	3.75^2^	[3.18^2^, 4.43^2^]	359.20	< 0.0001
Wong, Goh, and Ramachandran 2022	0.1529	[0.0786, 0.2763]	94.20%	[92.1%, 95.8%]	4.17^2^	[3.57^2^, 4.87^2^]	416.30	< 0.0001
Cañas et al. 2021	0.1638	[0.0836, 0.2960]	94.10%	[91.9%, 95.7%]	4.12^2^	[3.52^2^, 4.81^2^]	412.42	< 0.0001
Volkert et al. 2021	0.1452	[0.0768, 0.2575]	93.80%	[91.5%, 95.5%]	4.02^2^	[3.432, 4.71^2^]	384.55	< 0.0001
Inoue et al. 2021	0.1653	[0.0847, 0.2976]	94.10%	[92.0%, 95.7%]	4.12^2^	[3.53^2^, 4.82^2^]	412.49	< 0.0001
Kambanis et al. 2020	0.1796	[0.0964, 0.3099]	93.90%	[91.6%, 95.5%]	4.04^2^	[3.45^2^, 4.73^2^]	381.33	< 0.0001
Duncombe Lowe et al. 2019	0.1728	[0.0900, 0.3061]	93.60%	[91.2%, 95.4%]	3.97^2^	[3.38^2^, 4.65^2^]	381.93	< 0.0001
Lange et al. 2019	0.1723	[0.0905, 0.3033]	94.10%	[92.0%, 95.7%]	4.13^2^	[3.54^2^, 4.83^2^]	410.47	< 0.0001
Gray et al. 2018	0.1644	[0.0845, 0.2952]	94.20%	[92.1%, 95.8%]	4.15^2^	[3.55^2^, 4.85^2^]	415.67	< 0.0001
Nicely et al. 2014	0.1649	[0.0844, 0.2974]	94.10%	[91.9%, 95.7%]	4.11^2^	[3.52^2^, 4.81^2^]	411.69	< 0.0001

Abbreviations: CI—confidence interval; DF—degrees of freedom; LRT—likelihood ratio test; *Q*—Cochrane's *Q*.

**TABLE 5:** Jackknife sensitivity analysis for autism papers (*k* = 3).

Author excluded	Proportion	Proportion [95% CI]	*I* ^2^	*I* ^2^ [95% CI]	*H* ^2^	*H* ^2^ [95% CI]	LRT *Q*	LRT *Q*; p
Koomar et al. 2021	0.0807	[0.0113, 0.4031]	97.70%	[94.3%, 99.0%]	6.54^2^	[4.18^2^, 10.22^2^]	37.78	< 0.0001
Nygren et al. 2021	0.0707	[0.0126, 0.3112]	98.50%	[96.8%, 99.3%]	8.26^2^	[5.60^2^, 12.16^2^]	161.49	< 0.0001
Sedgewick, Leppanen, and Tchanturia 2020	0.2106	[0.1998, 0.2219]	29.90%	N/A	1.19^2^	N/A	1.34	< 0.2462

Abbreviations: CI—confidence interval; DF—degrees of freedom; LRT—likelihood ratio test; NA—not available; *Q*—Cochrane's *Q*.

### 3.5 Meta‐Regression Models

Univariate meta‐regression models identified that gender presentation (residual Tau^2^ = 1.09; *p* = 0.0008***) and ethnicity (residual Tau^2^ = 0.7421; *p* = 0.0001***) served as significant sources of heterogeneity across included studies (Table S2). Studies with a higher proportion of male individuals with ARFID as well as studies with Australian relative to non‐western participants reported higher co‐occurrence with autism. Multivariate assessment of predictors identified that ARFID sample size, gender, and ethnicity were variables serving as significant sources of heterogeneity. Larger sample sizes and studies with participants from Europe relative to non‐western participants resulted in a lower prevalence of co‐occurrence, while male‐predominant publications and studies with participants from the UK relative to non‐western participants identified a higher prevalence of co‐occurrence. As meta‐regression is considered appropriate when assessing 10 or more studies (Cumpston et al. 2019), models could not be conducted for publications with Autistic cohorts due to the limited available data (*k* = 3).

## 4 Discussion

This meta‐analysis included 21 studies with 7442 individuals, reporting significant co‐occurrence between ARFID and autism. Findings identified an autism diagnostic prevalence of 16.27% in those with ARFID. Whilst we were unable to directly compare these prevalence values to the general population in this meta‐analysis, these values appear to be significantly elevated, that is, they are more than 15 times higher than prevalence estimates of autism within the general population (Zeidan et al. 2022). Importantly, the prevalence of autism diagnoses has increased over the past couple of decades (Chiarotti and Venerosi 2020; Russell et al. 2022), particularly in children, where prevalence has increased by 140.3% in the past decade within the United Kingdom (McConkey 2020) and 250% in Europe (Chiarotti and Venerosi 2020). Increased rates of prevalence are associated with changes in classification criteria alongside heightened awareness of autism and associated Autistic characteristics (Hansen, Schendel, and Parner 2015). Current global rates are relatively similar across meta‐analyses, calculated at 1.00% (Zeidan et al. 2022), 0.72% (Talantseva et al. 2023), and 0.6% (Salari et al. 2022). ARFID publications included for this review exhibited significant heterogeneity in terms of study settings, means of diagnosis or evaluation and sample size, and subsequent rates of co‐occurrence with autism varied from 1.96% to 88.00%, which is a significantly large range of estimates. However, these estimates reflect the wide range of ARFID prevalence values identified in literature, ranging from 0.3% to 84.0% (Sanchez‐Cerezo et al. 2023). Sources of heterogeneity were associated with participant gender and ethnicity, which reflect previously reported differences in autism diagnostic prevalence according to gender (Zeidan et al. 2022), and ARFID diagnostic prevalence in relation to ethnic background (D'Adamo et al. 2023). Heterogeneity in methodological approach is present in the vast majority of epidemiological ARFID research (Sanchez‐Cerezo et al. 2023), which is likely due to the novelty of the ARFID diagnostic criteria relative to other FEDs and EDs. In addition to high levels of heterogeneity, the significance of Egger's test values and corresponding funnel plot asymmetry point toward the presence of publication bias influencing the prevalence of co‐occurrence. Findings from this meta‐analysis suggest that further research investigating rates of ARFID across larger sample sizes, more diverse ranges of ethnic backgrounds as well as across gender would benefit our understanding of ARFID prevalence as well as it's co‐occurrence with autism.

Findings also report a rate of 11.41% ARFID diagnoses or trait presence in Autistic groups. However, it is important to interpret this with caution, due to the limited literature available evaluating rates of ARFID in autism research as well as the high level of heterogeneity in reported findings. Importantly, all included autism studies were conducted in population‐based study settings, which may explain the lower rate of co‐occurrence seen in Autistic groups relative to groups with ARFID. When considering prevalence of ARFID estimated via population‐based means, our identified rate of co‐occurrence is significantly elevated in comparison to lower estimates of ARFID prevalence (e.g., 0.3%; Chen et al. 2020; Sanchez‐Cerezo et al. 2023), and very similar to higher estimates (e.g., 15.5%; Gonçalves et al. 2019; Sanchez‐Cerezo et al. 2023). Despite extant literature reporting relatively similar autism prevalence values, rates of ARFID in publications with Autistic groups highly varied, ranging from 2.10% to 28.00%, which is likely attributed to the heterogeneity associated with ARFID identification and screening. Unlike the relatively stable reports of autism diagnostic prevalence within the general population, prevalence values of ARFID vary extensively and are unreliable to determine a consensus on estimates. Findings from this work highlight a significant need to conduct research investigating rates of ARFID in Autistic cohorts, as well as to explore potential distinctions in rates of co‐occurrence according to gender. While method of autism diagnosis, such as clinical diagnosis versus questionnaire‐based screening, was not considered a significant source of heterogeneity associated with determining rates of co‐occurrence, it is important for researchers and clinicians to consider implementing autism identification measures free from gender bias (Burrows and Zheng 2024).

The high imprecision in the prevalence estimates associated with ARFID highlights the necessity toward implementing clear guidelines to standardize or operationalize ARFID DSM‐5 diagnostic criteria and screening measurements. For instance, only the ARFID module of the ED Examination (Schmidt et al. 2019) and the Pica, ARFID and Rumination Disorder Interview (PARDI; Bryant‐Waugh et al. 2019) are structured clinical interviews validated for examination of ARFID, and there is no clear ‘gold standard’ self‐report measure among existing tools such as the NIAS (Zickgraf and Ellis, 2018), self‐report version of the PARDI (Bryant‐Waugh et al. 2022), Food Neophobia Scale (Pliner and Hobden 1992), and Eating Disturbances in Youth Questionnaire (EDY‐Q; Kurz et al. 2015) (Kambanis and Thomas 2023). This meta‐analysis included publications using a variety of tools to assess ARFID, such as the child version of the EDE‐Q's ARFID module (Brosig et al. 2023; Bertrand et al. 2024), the PARDI (Kambanis et al. 2020; Watts et al. 2023), the NIAS (Koomar et al. 2021), and the ARFID‐Brief Screener (Dinkler et al. 2022) outside of general use of the DSM‐5 criteria. Aside from existing methodological differences presented by sample size, participant age, and geographical location, prevalence values can so much as double depending on how the DSM‐5 criteria for ARFID is implemented in research or diagnosis, such as how strictly existing criteria are defined or many criteria are incorporated to diagnose ARFID (Harshman et al. 2021). While the exploratory meta‐regression analysis did not identify method of ARFID diagnosis as a significant source of heterogeneity when determining autism co‐occurrence rates in ARFID, the standardization of ARFID screening tools and diagnostic measurements are crucial toward understanding the impact of comorbidities or co‐occurring conditions associated with the FED.

A burgeoning field of research focuses on the overlap between autism and AN (Westwood et al. 2018; Tchanturia et al. 2019), and findings from this meta‐analysis highlight the necessity for investigations of this overlap to include the ARFID diagnosis. Current recommendations for the co‐occurrence of autism and AN highlight the need to implement autism screening measures within clinical and health services, as well as to provide adaptable ED interventions tailored to the individual (Tchanturia 2022). An important consideration when observing the limited amount of literature evaluating rates of ARFID in Autistic cohorts are challenges associated with when to identify or screen for ARFID in Autistic populations. Certain features of ARFID, such as selective eating and limited dietary diversity, are often observed in autism (Baraskewich et al. 2021), and thus clinicians may risk either incorrectly diagnosing Autistic individuals with ARFID, or overlooking the ARFID diagnosis entirely. To protect Autistic preferences and identity as well as prioritize health and wellbeing, we recommend screening for ARFID when an individual shows signs of poor mental and/or physical health as reflected within the existing DSM‐5 criteria. Elevated rates of co‐occurrence between autism and ARFID reported in this meta‐analysis not only suggest operationalization autism screening in clinical ED services, but also recommend the implementation of ARFID screening measures in Autistic individuals who require support for pathological disordered eating.

### 4.1 Limitations

This systematic review is not without limitations. First, meta‐analysis of included publications identified significant rates of heterogeneity across studies. This may result from the relative recent nature of the ARFID diagnostic criteria relative to other FEDs, and the subsequent novelty of measures used for its classification. Heterogeneity may also result from differences in reported rates of co‐occurrence that may be due to participant age, methodological approach, objectives associated with studies (e.g., ED intervention, retrospective chart review, clinical drug trial, etc.), geographical location of the study and sample population (e.g., clinical ED versus population‐based). Due to the relative novelty of the ARFID diagnosis and subsequent limited nature of available data, this meta‐analysis did not exclude studies with small sample sizes (e.g., Gray et al. 2018—*n* = 14; Wong, Goh, and Ramachandran 2022—*n* = 12). In addition to existing methodological heterogeneity across included publications, these small‐sample studies may have contributed toward an increased likelihood of publication bias impacting prevalence of co‐occurrence between ARFID and autism, potentially inflating existing proportions seen in this meta‐analysis.

A large majority of included studies focused on child and adolescent populations, and findings relating to co‐occurrence between ARFID and autism are not generalizable across the age range. In line with limitations brought on by unavailability of diverse age ranges, there was insufficient information to conduct a meta‐regression or subgroup analysis according to sample type for this study. The majority of publications recruited samples from clinical ED settings (*k* = 17/21), which are ultimately not representative of the ARFID population, and particularly the Autistic population. While those with ARFID, and particularly Autistic individuals, often present to ED services, they also present to a diverse range of other services, such as general mental health services, general health and pediatric services, occupational therapy and dietetics services, and such individuals are not included in studies conducted in ED clinic environments (Duffy et al. 2024). It is also important to acknowledge the potential for Berkson's bias in identified prevalence values of co‐occurrence, in which the association of variables being studied affect the inclusion or selection of study subjects (Westreich 2012). As individuals with ARFID or Autistic individuals are more likely to be referred to clinical settings, studies incorporating clinical ED samples may subsequently overestimate co‐occurrence between both conditions. Across extant literature, there is a relatively large difference between clinical ED and population‐based rates, ranging from 5.0% to 22.5% and 0.3% to 15.5%, respectively (Sader et al. 2023; Sanchez‐Cerezo et al. 2023), and future research should incorporate evaluation of estimates according to study environment.

Additionally, the majority of studies focusing on ARFID and/or autism are restricted to few locations, predominantly western countries (e.g., US, UK). Many included publications (*k* = 11/21) did not specify the ethnic or racial distribution of participants, and publications that did include ethnic distribution focused on predominantly Caucasian/white participant samples (61.64% Caucasian/white), which limits the generalizability of these findings to global, non‐western populations. Additionally, previous population‐based research report conflicting findings as to whether there are significant differences in the presence of ARFID symptomatology according to ethnicity (D'Adamo et al. 2023; Sader et al. 2023). While some publications in this meta‐analysis exclusively focused on ARFID in multiethnic (Nygren et al. 2021) or non‐white/Caucasian (Inoue et al. 2021; Wong, Goh, and Ramachandran 2022) samples, further is research warranted in non‐western countries and across ethnicities to further understand prevalence across geographical location and ethnic background.

Importantly, this meta‐analysis was unable to statistically evaluate comparator values in the form of odds ratios or the rates of co‐occurrence between autism and ARFID according to distinct ARFID profiles. The limited availability of data for ARFID profiles is likely due to the novelty of the formal introduction of the LOF, SS, and FOC profiles to the text revision of the DSM‐5, which occurred in 2022. Future research evaluating specific ARFID profiles is highly warranted, with preliminary evidence seen in this study noting an elevated autism prevalence in those with the SS ARFID profile (Katzman et al. 2022; Sanchez‐Cerezo et al. 2024). In addition to this preliminary evidence, future research should incorporate investigation of autism co‐occurrence within “combined” ARFID profile presentations, with SS and LOF symptoms strongly co‐occurring within a proportion of ARFID patients (Abber et al. 2024; Sanchez‐Cerezo et al. 2024).

## 5 Conclusion

This study is the first meta‐analysis statistically evaluating the co‐occurrence of ARFID and autism, identifying significant rates of ARFID prevalence in Autistic groups, and autism diagnostic prevalence in those with ARFID. The co‐occurrence between ARFID and autism is significantly elevated relative to general population‐based rates of autism diagnoses, as well as the majority of estimates relating to population‐based rates of ARFID. In studies with ARFID cohorts, estimates of prevalence were significantly heterogeneous according to gender and ethnic background. There was insufficient data to explore sources of heterogeneity in studies with Autistic groups, highlighting a need to interpret ARFID prevalence findings in Autistic groups with caution as well as the paucity of assessment for ARFID in this community. Findings from this work firstly suggest an increased proportion of research geared towards ARFID across ethnic backgrounds and geographical location, as well as increased research screening for ARFID in studies with Autistic cohorts. Additionally, estimates provided by this meta‐analysis warrant a diversion of resources and funding toward 1. Standardization of ARFID diagnosis and screening procedures, including the addition of guidelines to support clinicians, 2. Means to screen for autism in clinical ED settings, and 3. The implementation of tailored and individualized treatment for Autistic individuals with ARFID.

## Author Contributions

**Michelle Sader:** conceptualization, data curation, formal analysis, investigation, methodology, resources, software, supervision, visualization, writing – original draft, writing – review and editing. **Annabel Weston:** investigation, methodology, writing – review and editing. **Kyle Buchan:** conceptualization, investigation, methodology. **Jess Kerr‐Gaffney:** methodology, writing – review and editing. **Karri Gillespie‐Smith:** conceptualization, funding acquisition, writing – review and editing. **Helen Sharpe:** funding acquisition, investigation, methodology, writing – review and editing. **Fiona Duffy:** conceptualization, funding acquisition, investigation, methodology, project administration, supervision, writing – review and editing.

## Conflicts of Interest

The authors declare no conflicts of interest.

## Supporting information


Table S1.
Table S2.
Figure S1.

## Data Availability

The data that support the findings of this study are available from the corresponding author. All raw extracted data, processed data and code used to conduct the meta‐analysis have been made available via the following Open Science Framework link (https://osf.io/PBV6K).
