# Supplementary material for: The Co‐Occurrence of Autism and Avoidant/Restrictive Food Intake Disorder (ARFID): A Prevalence‐Based Meta‐Analysis
Source: Int J Eat Disord. 2025 Jan 6;58(3):473–88. doi: 10.1002/eat.24369 (PMC11891632; doi:10.1002/eat.24369)
Supplement: Supplementary file 1 — Table S1. Table S2. Figure S1. [file EAT-58-473-s001.docx]

**The Co-occurrence of Autism and Avoidant/Restrictive Food Intake Disorder (ARFID): A Prevalence-Based Meta-Analysis – Supplementary Material**

| **Table S1: Quality assessment of publications included for meta-analysis using the JBI Critical Appraisal Checklist for prevalence-based studies** | | | | | | | | | |  |
| --- | --- | --- | --- | --- | --- | --- | --- | --- | --- | --- |
| **Included Papers** | **JBI CA Item 1:**  *Was the sample frame appropriate to address the target population?* | **JBI CA Item 2:**  *Were study participants sampled in an appropriate way?* | **JBI CA Item 3:**  *Was the sample size adequate? ^a^* | **JBI CA Item 4:**  *Were the study subjects and the setting described in detail? ^b^* | **JBI CA Item 5:**  *Was the data analysis conducted with sufficient coverage of the identified sample?* | **JBI CA Item 6:**  *Were valid methods used for the identification of the condition?* | **JBI CA Item 7:**  *Was the condition measured in a standard, reliable way for all participants?* | **JBI CA Item 8:**  *Was there appropriate statistical analysis?* | **JBI CA Item 9:**  *Was the response rate adequate, and if not, was the low response rate managed appropriately?* | |
| Bertrand et al., 2024 | No | Yes | Yes | No | Yes | Yes | Yes | Yes | NA^c^ | |
| Sanchez-Cerezo et al., 2024 | No | Yes | Yes | Yes | Yes | Yes | Yes | Yes | NA^c^ | |
| Brosig et al., 2023 | No | Yes | Yes | Yes | Yes | Yes | Yes | Yes | NA^c^ | |
| Chatoor et al., 2023 | No | Yes | No | No | Yes | Yes | Yes | Yes | NA^c^ | |
| Watts et al., 2023 | No | Yes | Yes | No | Yes | Yes | Yes | Yes | NA^c^ | |
| Taylor et al., 2022 | No | Yes | No | Yes | Yes | Yes | Yes | Yes | Yes | |
| Dinkler et al., 2022 | Yes | Yes | Yes | Yes | Yes | Yes | Yes | Yes | Yes | |
| Farag et al., 2022 | No | Yes | Yes | Yes | Yes | Yes | Yes | Yes | Unclear | |
| Katzman et al., 2022 | No | Yes | No | Yes | Yes | Yes | Yes | Yes | NA^c^ | |
| Wong et al., 2022 | No | Yes | No | No | No | Yes | Yes | Yes | NA^c^ | |
| Cañas et al., 2021 | No | Yes | Yes | No | Yes | Yes | Yes | Yes | NA^c^ | |
| Volkert et al., 2021 | No | Yes | No | Yes | Yes | Yes | Yes | Yes | NA^c^ | |
| Koomar et al., 2021 | Yes | Yes | Yes | Yes | Yes | Yes | Yes | Yes | NA^c^ | |
| Nygren et al., 2021 | Yes | Yes | No | No | Yes | Yes | Yes | Yes | Unclear | |
| Inoue et al., 2021 | No | Yes | Yes | Yes | Yes | Yes | Yes | Yes | Unclear | |
| Kambanis et al., 2020 | Yes | Yes | Yes | Yes | Yes | Yes | Yes | Yes | Unclear | |
| Sedgewick et al., 2020 | Yes | Yes | Yes | Yes | Yes | Yes | Yes | Yes | Unclear | |
| Lowe et al., 2019 | No | Yes | Yes | Yes | No | Yes | No | Yes | NA^c^ | |
| Lange et al., 2019 | No | Yes | No | Yes | No | Yes | Yes | Yes | NA^c^ | |
| Gray et al., 2018 | No | No | No | No | Yes | Yes | Yes | Yes | NA^c^ | |
| Nicely et al., 2014 | No | Yes | Yes | Yes | Yes | Yes | Yes | Yes | NA^c^ | |

**Table S1.** ^a^ – Adequacy of sample size was measured using the following equation: n=Z2p(1-p)/d2 (n – sample size; Z – standard normal distribution at a desired confidence interval; p – expected prevalence or proportion; d – precision, corresponding to effect size).

^b^ – Publications were ranked as not meeting item 4 criteria if they were missing information on participant ethnicity.

^c^ – Response rate was not applicable for retrospective chart reviews.

[Abbreviations: CA – Critical Appraisal; JBI – Joanna Briggs Institute; NA – Not Applicable


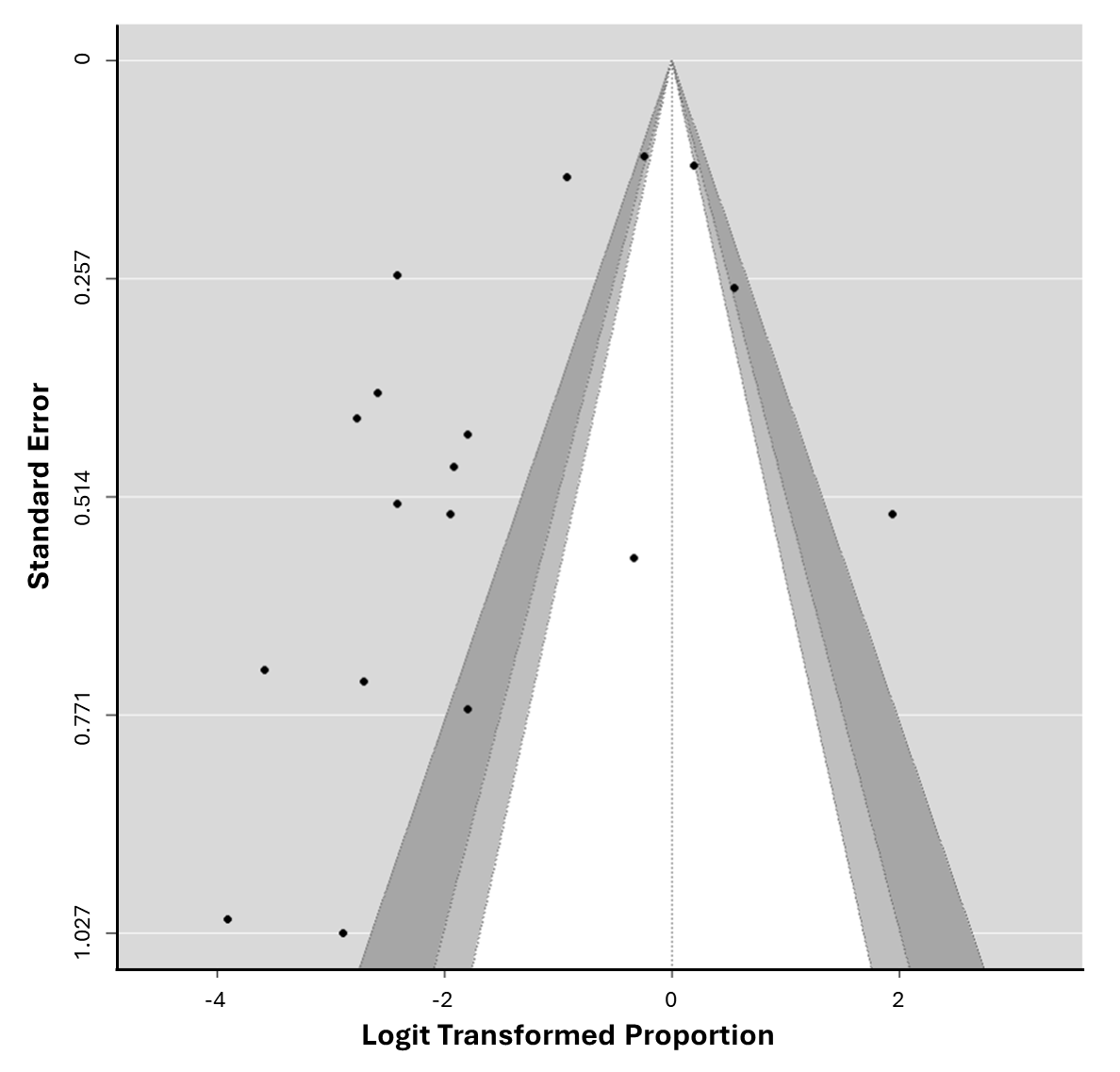
**Figure S1.** A contour-enhanced funnel plot depicting autism diagnostic prevalence rates in studies with ARFID groups (k=18). Light and dark grey regions correspond to p=0.1-0.05 and p=0.5-0.01 respectively, with the white region corresponding to p>0.1. The extensive representation of publication effect sizes lying outside of the dark grey regions suggests a high likelihood of publication bias.

| **Table S2. Results of univariate and multivariate mixed-effects meta-regression models for all ARFID publications (k=18)** | | | | | | | | | |
| --- | --- | --- | --- | --- | --- | --- | --- | --- | --- |
| **Factor** |  | **No. of Studies** | **Coefficient Estimate** | **CI** | **Model p** | **Tau^2^ Residual Heterogeneity** | **I^2^ Residual Heterogeneity** | **LRT** | **LRT p** |
| Date of Publication | | 18 | 0.1193 | [-0.1782, 0.4168] | 0.4318 | 2.0560 | 96.12% | 381.1927 | <0.0001*** |
| Study Environment | | 18 | -1.6646 | [-3.8784, 0.5492] | 0.1405 | 1.8726 | 96.15% | 369.202 | <0.0001*** |
| Method of ARFID Diagnosis | | 18 | -1.8046 | [-4.0928, 0.4835] | 0.1221 | 1.8784 | 96.16% | 379.1147 | <0.0001*** |
| Method of Autism Diagnosis^a^ | | 17 | N/A | N/A | N/A | N/A | N/A | N/A | N/A |
| ARFID Sample Size | | 18 | 0.0041 | [-0.0030, 0.0112] | 0.2552 | 1.4081 | 95.97% | 321.0649 | <0.0001*** |
| Total Study Sample Size | | 18 | -0.0002 | [-0.0010, 0.0007] | 0.7063 | 2.1204 | 96.53% | 417.2137 | <0.0001*** |
| Gender (% Male) | | 16 | 0.0483 | [0.0200, 0.0767] | 0.0008*** | 1.0888 | 93.69% | 128.9222 | <0.0001*** |
| Mean Age | | 15 | -0.1172 | [-0.2822, 0.0478] | 0.1640 | 1.9829 | 95.33% | 272.5011 | <0.0001*** |
| Ethnicity^b^ | *Western (AUS)* | 1 | 3.6942 | [1.3786, 6.0097] | 0.0018**^c^ | 0.7421 | 91.09% | 146.0089 | <0.0001*** |
|  | *Western (CAN)* | 1 | -0.7681 | [-2.8768, 1.3405] | 0.4752^c^ |  |  |  |  |
|  | *Western (EUR)* | 4 | -1.0823 | [-2.6535, 0.4889] | 0.1770^c^ |  |  |  |  |
|  | *Western (UK)* | 3 | 1.3428 | [-0.1784, 2.8640] | 0.0836^c^ |  |  |  |  |
|  | *Western (US)* | 6 | -0.3041 | [-1.7229, 1.1147] | 0.6744^c^ |  |  |  |  |
| Multivariate^d^ | Date of Publication | 15 | -0.1656 | [-1.1552, 0.2480] | 0.2480 | 0.0 | 0.00% | 6.4485 | 0.0398* |
|  | Study Environment |  | -1.3658 | [-3.0872, 0.3557] | 0.1199 |  |  |  |  |
|  | Method of ARFID Diagnosis |  | -1.7717 | [-3.8614, 0.3180] | 0.0996 |  |  |  |  |
|  | ARFID Sample Size |  | -0.0128 | [-0.0202, -0.0054] | 0.0007*** |  |  |  |  |
|  | Total Sample Size |  | 0.0003 | [-0.0006, 0.0012] | 0.5270 |  |  |  |  |
|  | Gender (% Male) |  | 0.0541 | [0.0287, 0.0794] | <0.0001*** |  |  |  |  |
|  | Mean Age |  | -0.0984 | [-1.1236, 2.1823] | 0.1771 |  |  |  |  |
|  | Ethnicity: Western (AUS) |  | 0.5393 | [-1.1236, 2.1823] | 0.5302 |  |  |  |  |
|  | Ethnicity: Western (CAN) |  | 1.0167 | [-0.6092, 2.6426] | 0.2203 |  |  |  |  |
|  | Ethnicity: Western (EUR) |  | -1.9323 | [-3.2578, -0.6069] | 0.0043** |  |  |  |  |
|  | Ethnicity: Western (UK) |  | 2.6924 | [0.7248, 4.6600] | 0.0073** |  |  |  |  |
|  | Ethnicity: Western (US) |  | -1.2645 | [-2.7605, 0.2315] | 0.0976 |  |  |  |  |

**Table S2.** * – p<0.05; ** – p<0.01; *** – p<0.001

^a^ – All publications utilised clinical interviews, professional diagnoses or previous medical history to diagnose autism and no contrasts could be generated

^b^ – Non-Western is used as a reference level to compare between different ethnic groups

^c^ – p=0.0001 for the entire sample

^d^ – Method of Autism Diagnosis excluded from the multivariate model

[Abbreviations: ARFID – Avoidant/restrictive food intake disorder; AUS – Australia; CAN – Canada; CI – Confidence Interval; EUR – Europe; LRT – Likelihood Ratio Test; N/A – Not applicable; No. – Number; UK – United Kingdom; US – United States]
